# A Novel Strategy for Mixed Jam Evaluation: Apparent Indicator, Sensory, Metabolomic, and GC-IMS Analysis

**DOI:** 10.3390/foods13071104

**Published:** 2024-04-03

**Authors:** Ruxianguli Maimaitiyiming, Huimin Zhang, Jiayi Wang, Liang Wang, Lei Zhao, Bingze Liu, Keping Chen, Aihemaitijiang Aihaiti

**Affiliations:** 1 School of Life Science and Technology, Xinjiang University, Urumqi 830046, China; rm725276492@126.com (R.M.); 15065643133@163.com (H.Z.); nonthermal_jyw@163.com (J.W.); wl1390593786@163.com (L.W.); zl15049967606@163.com (L.Z.), liubz0831@163.com (B.L.); 2 Xinjiang Huize Food Limited Liability Company, Urumqi 830046, China; clnb188@126.com

**Keywords:** mixed jam, untargeted metabolomics, volatile aroma components

## Abstract

Jam is a popular traditional and modern food product for daily consumption. However, the benefits of mixed jams over single-fruit jams have not been thoroughly explored, with analyses limited to superficial indices. In this study, Xinjiang special *Morus nigra* L. and *Prunus domestica* L. were used as raw materials to prepare single-fruit and mixed jams, and their differences in antioxidants, organoleptic qualities, pH, texture, and color were analyzed. The dynamics of metabolites before and after thermal processing were assessed using untargeted metabolomics. The results indicate that the main metabolites were flavonoids, terpenoids, amino acids, phenolic acids, and carbohydrates. Flavonoid metabolites changed significantly after thermal processing, with 40 up-regulated and 13 down-regulated. During storage, polyphenols were the prominent differential metabolites, with fifty-four down-regulated and one up-regulated. Volatile aroma components were analyzed using gas chromatography–ion mobility spectrometry (GC-IMS); the aroma components E-2-hexenal, E-2-pentenal, 3-methylbutanal, 1-penten-3-ol, tetrahydro-linalool, 1-penten-3-one, hexyl propionate, isoamyl acetate, α-pinene, and propionic acid in mixed jam were significantly higher than in single-fruit jam. In this study, untargeted metabolomics and GC-IMS were used to provide a more comprehensive and in-depth evaluation system for jam analysis.

## 1. Introduction

Jam-making is one of the oldest food preservation processes [1]. It is prepared by boiling fruit pulp with sucrose, pectin, acids, and other ingredients such as preservative, coloring, and flavoring agents and is widely consumed worldwide [2,3]. With the increasing demand for new flavors products, mixed jams are gradually becoming a popular food in daily diets. However, the advantages of mixed jams compared with single-fruit jams have not been explored in depth, with existing studies focused on epigenetic indices such as sensory, textural, and nutrient contents.

The field of metabolomics, which is an integral component of systems biology, focuses on the comprehensive analysis of small molecule metabolites and their dynamic alterations within living organisms, specific tissues, and individual cells [4]. Metabolites include a wide range of compounds such as sugars, amino acids, organic acids, secondary metabolites (e.g., alkaloids and flavonoids), and lipids [5]. In recent years, metabolomics has been widely used for food component analysis. Panseri and Arioli [6] estimated global metabolomic changes in irradiated chicken, turkey, and mixed (chicken, turkey, and pork) stranded meats to assess possible food safety concerns regarding metabolomic alterations. At present, 402 metabolites were identified and it was found that irradiation did not pose any safety concerns. Wang and Wei [7] investigated metabolites in *Lactobacillus plantarum* L3-fermented milk using metabolomic and peptidomic analyses; the metabolites included Thr-Pro, Val-Lys, l-creatine, pyridoxine, and cytosolic acid, which could improve milk flavor. There are also studies revealing commonalities and differences in wines made with three different grape varieties by a combination of UPLC-Orbitrap-MS/MS analysis and the sensory analysis of untargeted metabolites [8].

Gas chromatography–ion mobility spectrometry (GC-IMS) is a new method for flavor analysis that has developed rapidly in recent years. This method has high separation efficiency and does not require sample pre-treatment. As a coupled technique, GC-IMS has the advantages of rapid detection speed and a large volume of processed information [9]. In addition, it is also applied to food processing monitoring, food identification and classification, detection of chemical reagents, adulteration detection, evaluation of food freshness and spoilage, and evaluation of aroma changes during food storage, among other uses [10,11,12].

*Prunus domestica* L. and *Morus nigra* L. are widely used in the food, pharmaceutical, and cosmetic industries, and are major cash crops in Xinjiang [13]. However, *Prunus domestica* L. is a leapfrog fruit with rapid fruit ripening, which limits its shelf life and suitability for fresh consumption [14]. Similarly, the fragile texture of blackberry (*M. nigra* L.) also limits its use for fresh consumption. The processing of *P. domestica* L. and *M. nigra* L. is an effective means to minimize postharvest losses and increase nutritional values. The aim of this study was to integrate metabolism and GC-IMS to provide a more comprehensive and in-depth evaluation system for jam analysis based on the traditional analytical strategy using *P. domestica* L. and *M. nigra* L. mixed jams as experimental subjects. The results can serve as a foundation for assessing mixed jam. For instance, non-targeted metabolomics and GC-IMS were employed to explore the impact of varying fruit addition ratios on metabolites and aroma compounds in intricate jams. Furthermore, this study has the potential to enhance the value of added fruits, thereby offering consumers healthier and more appealing jam products.

## 2. Materials and Methods

### 2.1. Materials

Frozen *M. nigra* L. and dried *P. domestica* L. were purchased at a local market at Urumqi, Xinjiang. Methanol and ethanol were obtained from Tianjin Beilian Fine Chemicals Development Co. (Tianjin, China). Methanol and acetonitrile were provided by Micron Co. (Boise, ID, USA). The hydroxyl radical scavenging rate kit was purchased from Beijing Box Biotechnology Co. (Beijing, China). The total antioxidant capacity kit was purchased from Nanjing Jiancheng Bioengineering Institute (Nanjing, China).

### 2.2. Mixed Jam Preparation

**Figure 1:**
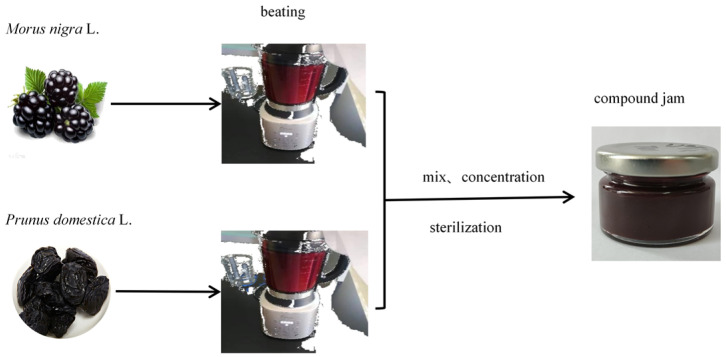
The process of making *Morus nigra* L. and *Prunus domestica* L. mixed jam.

*M. nigra* L. and intact dried *P. domestica* L. with moderate maturity, and free from damage and disease, were selected; dried *P. domestica* L. was rehydrated, de-cored, and set aside. The raw materials were pulped in a pulper according to a material-to-water ratio of 1:1. The pulp of raw materials was added according to a ratio of *M. nigra* L. pulp to *P. domestica* L. pulp 1:4, 2:3, 3:2, or 4:1 (*w*/*w*). The raw fruit accounted for 84.4% of the total ingredients; sugar accounted for 15% of the total ingredients, and a thickening agent (pectin) accounted for 0.6% of the total ingredients. Subsequently, the jam was heated and concentrated at a temperature of 80–100 °C, and the jam was ready to be released from the pot when its soluble solids content was 38% ± 2%. Immediately after release from the pot, the jam was packed in jars while hot, sealed and sterilized in a water bath at 80 °C for 30 min [15], and then cooled to room temperature around 25 °C. The production process is shown in Figure 1.

### 2.3. Physical and Chemical Indicators Analysis

The soluble solids content of the jam was determined using a hand-held glucometer. The pH value was determined using a pH meter after adding an equal amount of water to the jam and mixing well. Total acid was determined through potentiometric titration using a pH meter with reference to the National Standard for Food Safety Determination of Total Acid in Food [16]. The color of the samples was determined using a colorimeter (CHN Spec CS-200, CHN Spec Technology Co., Ltd., Hangzhou, China), which was calibrated using a standard white plate before use [1]. Texture was determined using a texture meter (TA.XTC-18; Bao Sheng, Shanghai, China) with a cylindrical flat-tipped probe with a diameter of 36 mm (model TA/36) and a 500 N force transducer. Jam samples were placed in a glass dish with a constant thickness and underwent two 6 mm compression cycles (speed 0.2 mm/s) [17]. Force–time curves were obtained using BosinTechTA (1.2.6.0) software. The values of texture characterization parameters, including hardness, adhesiveness, chewiness, and cohesiveness, were obtained.

### 2.4. Polyphenols and Antioxidants

The polyphenol content was determined according to established methods [18], with modification. Briefly, a 2 g sample of jam was weighed and 80% methanol was added for grinding. After grinding, 80% methanol solution was used to fix the volume to 30 mL in a centrifuge tube. An ultrasonic water bath was performed for 20 min, followed by centrifugation at 11,000× *g* at 4 °C for 10 min, and the supernatant was collected. The extracted supernatant (50 μL) and Folin reagent (250 μL) were added to 3 mL of distilled water rests for 6 min. Then, 750 μL of 20% sodium carbonate solution was added, and the sample was incubated for 90 min at room temperature with light protection. The absorbance was measured at 765 nm, and the polyphenol content was calculated according to the standard curve; the results were expressed as mg gallic acid equivalent/100 g fresh fruit weight (mg GAE/100 g FW).

The total antioxidant capacity was determined according to the instructions of the corresponding kit (A015-1-2; Nanjing Jiancheng Bioengineering Research Institute, Nanjing, China); the hydroxyl radical scavenging rate was determined according to the instructions of the corresponding kit (AKAO013; Beijing Box Bioengineering Technology Co., Ltd., Beijing, China).

To screen the antioxidant activity, we performed DPPH free radical scavenging according to previously published methods [19], with modifications. Briefly, 100 μL of supernatant diluted 25-fold with 60% ethanol (9500 rpm centrifugation for 5 min) and 100 μL of DPPH-ethanol solution (0.2 mmol/L) were added to the 96-well plate and were mixed and shaken well. The reaction was carried out at room temperature and protected from light for 30 min, and the measured absorbance values were determined at 517 nm [15].

To further evaluate the antioxidant activity, ABTS radical scavenging was performed according to previously published methods [20], with modifications. Briefly, 30 μL (9500 rpm centrifugation for 5 min) of 60% sample solution was mixed with 210 μL of ABTS solution (7 mmol/L ABTS with 2.45 mmol/L potassium persulfate at a 1:1 ratio to form a working solution, diluted with anhydrous ethanol to an absorbance value of 0.7 ± 0.02), and it was mixed and shaken well. The reaction was carried out at room temperature and protected from light for 30 min, and the absorbance value was measured at 734 nm.

### 2.5. Sensory Evaluation

A panel of nine food professionals with experience in sensory evaluation was formed, and the indicators were evaluated, scored, and averaged. The sensory attributes of the samples that were evaluated included the following: spreadability, odor, texture, color, and taste [21]. The panel members were asked to observe and taste each of the coded samples. After evaluating each sample, they rinsed their mouths with drinking water to avoid flavor interference [22]. The rating scale is shown in Table 1.

### 2.6. Metabolite Extraction and Analysis by Liquid Chromatography–Mass Spectrometry

The total amount of colonies, fungi, yeast, Escherichia coli, sensory properties, and soluble solids in the jam during storage were determined using the accelerated shelf-life test method [23]. According to the findings, the jam had a shelf life of 143 days at 20 °C for commercial storage and 25 days at 37 °C. During the whole storage period, no microbiological indications were found. In order to show the variability of the samples, the sample selected for the metabolic group was jam stored at 37 °C, the metabolome sampling nodes were taken every 10 d of storage, and the sampling endpoint was 25 d of storage. Metabolites were extracted from 100 mg of sample in a 2 mL centrifuge tube with a 6 mm diameter grinding bead. Subsequently, 800 μL of extraction solution (methanol/water = 4:1 [*v*/*v*]) containing four internal standards (L-2-chlorophenylalanine [0.02 mg/mL]) was used for metabolite extraction. Sample solutions were ground in a frozen tissue grinder for 6 min (−10 °C, 50 Hz), followed by low-temperature ultrasonic extraction for 30 min (5 °C, 40 kHz). The samples were allowed to stand at −20 °C for 30 min, followed by centrifugation for 15 min (4 °C, 13,000× *g*) [5]. The supernatant was pipetted into an injection vial with an internal cannula for analysis. An equal volume of metabolites from all samples was mixed to form a quality control (QC) sample, and one QC sample was inserted every 5–10 samples during the instrumental analysis to check the reproducibility of the whole analysis process.

Using a Waters BEH C18 column (100 mm × 2.1 mm; internal diameter, 1.7 µm), 3 μL of the sample was separated and detected through mass spectrometry. Mobile phase A was 2% acetonitrile in water (containing 0.1% formic acid) and mobile phase B was acetonitrile (containing 0.1% formic acid) [24]. The flow rate was 0.40 mL/min, and the column temperature was 40 °C. Subsequently, 3 μL of the sample was separated using a BEH Amide column (100 mm × 2.1 mm; internal diameter, 1.7 µm) and then detected through mass spectrometry. The mobile phase A was 95% acetonitrile water (containing 5 mM ammonium acetate), and mobile phase B was 5% acetonitrile water (containing 10 mM ammonium acetate) [25]. The flow rate was 0.40 mL/min, and the column temperature was 40 °C. The sample mass spectrometry signal acquisition was performed in positive and negative ion scanning mode, with a mass scanning range of 70–1050 *m*/*z* [25]. The ion spray voltage was 3500 V for positive ions and −3000 V for negative ions, with a sheath gas of 50 psi, an auxiliary heated gas of 13 psi, a heating temperature of 450 °C for the ion source, and a cyclic collision energy of 20–40–60 V. The resolution of the ion source was 70,000 for MS1 and 17,500 for MS2.

The stability of the model was assessed using principal component analysis (PCA) and orthogonal least squares discriminant analysis (OPLS-DA) with seven cycles of cross-validation [5]. The selection of significantly different metabolites was determined based on the variable weight (variable importance in projection [VIP]) values obtained from the OPLS-DA model and the *p*-value obtained from the student’s *t*-test. Metabolites with VIP > 1 and *p* < 0.05 were considered significantly different metabolites [24].

### 2.7. Analysis of Volatile Aroma Components

To prepare the sample, 2 g of jam was weighed in a 20 mL headspace bottle and then incubated at 60 °C for 20 min. Each sample was determined in three parallel groups. The injection volume was 500 µL, and non-shunt injection was used. The incubation speed was 500 rpm, and the injection needle temperature was 85 °C. The column temperature was 60 °C, and the carrier gas was high purity nitrogen (purity ≥ 99.999%). Boosting was programmed with an initial flow rate of 2.0 mL/min, which was held for 2 min and then linearly increased to 10.0 mL/min within 8 min, linearly increased to 100.0 mL/min within 10 min, and held for 30 min. The chromatographic runtime was 50 min [26]. The inlet temperature was 80 °C. The ionization source was tritium source (3H). The migration tube length was 53 mm, with an electric field strength of 500 V/cm, migration tube temperature of 45 °C, drift gas of high purity nitrogen (purity ≥ 99.999%), and flow rate of 75.0 mL/min [27]. The positive ion mode was used.

Targets were characterized using the GC retention index (NIST 2020) database and IMS migration time database search and comparison features in the VOCal (0.4.03) software. Plots of volatile constituents were generated using the Reporter, Gallery Plot, and Dynamic PCA plug-ins in the VOCal (0.4.03) data processing software for inter-sample comparison of VOCs. The detection limit is 10 ppb.

### 2.8. Data Processing and Statistical Analysis

The mean values among different groups were analyzed using one-way analysis of variance and the post hoc Duncan’s multiple range test in SPSS 26. A *p*-value of <0.05 was considered statistically significant. Each experiment was conducted independently three times.

## 3. Results and Discussion

### 3.1. Physical and Chemical Indicators

The pH is widely used as an important indicator for evaluating the quality of food products in the quality testing of various products. It plays an important role in enzyme activity, protein denaturation and microbial inactivation, and the pH should be lowered in order to improve the microbial stability of food products [28]. The experimental results demonstrate that the jam meets the national standard for jam [21]. The pH value of the jam ranged from 3.2 to 4.2 (Table 2). Both pH and total acid were related to the concentration of H^+^ when *Morus nigra* L. was present in jam. When the content increased, the pH value decreased significantly and the total acidity of the jam increased, indicating that *Morus nigra* L. provides a large amount of H^+^ for the jam system. Low pH reduces the negative charge of pectin molecules and then promotes hydrogen bonding of pectin molecules leading to precipitation [29]. The addition of sucrose as a component in jam-making at low pH favors the gel formation of high-methoxy pectin [29,30]. However, the pH of jam should not be too low, as this can lead to the deterioration of organoleptic qualities such as glucose crystallization, granular texture, excessively acidic flavor, and oozing.

The samples analyzed in the present study are shown in Table 3. ∆E represents the overall color difference between the sample and control groups, with larger ∆E values indicating a greater color difference. Using a whiteboard as a control, among the sample groups, the overall color difference compared to the control groups was significant only in the jam where the mass ratio of *M. nigra* L. to *P. domestica* L. added to the jam was 0:5. In contrast, the overall color difference in the rest of the groups was not significantly different compared to the control groups [22].

The brightness index L* represents the color brightness of the jam; a higher L* value corresponds to increased sample brightness and a lighter perceived color. In Table 3, it is observed that the brightness of the jam decreases with an increase in the proportion of *M. nigra* L. When the mass ratio is 1:4, 2:3, 3:2, and 4:1, there are no significant differences in brightness. The highest (31.85 ± 0.03) and lowest (28.14 ± 1.58) L* values were observed at *M. nigra* L. to *P. domestica* L. mass ratios of 0:5 and 5.0, respectively [31].

The a* index represents the red–green color spectrum of the object, with a positive value denoting red color and a negative value denoting green color. The a* value was positive for all sample groups, indicating a reddish coloration. Moreover, the values exhibited a trend of increasing and then decreasing, with the highest a* value (3.45 ± 0.11) observed at an *M. nigra* L. to *P. domestica* L. mass ratio of 2:3. This ratio resulted in the most intense red coloration of the jam. In contrast, at an *M. nigra* L. to *P. domestica* L. mass ratio of 5:0, the a* value was the lowest (2.28 ± 0.02), with the least intense red coloration of the jam [31].

The b* index represents the yellow–blue color spectrum of the object, with a positive value indicating yellow, and a negative value indicating blue. At an *M. nigra* L. to *P. domestica* L. mass ratio of 0:5, the b* value was positive, indicating yellowish coloration in the jam. For the other sample groups, the b* value was negative, indicating a bluish tone. Moreover, the red color of the jam tended to increase with the increase in *M. nigra* L. content [31].

The results presented in Table 4 illustrate the textural parameters of jams containing varying ratios of fruit ingredients. It was observed that the hardness, adhesion, and chewiness of the jams tended to decrease as the proportion of *M. nigra* L. increased. In contrast, no significant trend was observed for cohesion.

*P. domestica* L. has a high natural pectin content, and fresh plums are a rich source of dietary fiber and pectin. Plums were found to contain 0.6–1.5 g of dietary fiber, including 0.5–1.0 g of pectin, per 100 g of fresh pulp [32]. The hardness, adhesion, and chewiness of the jam increased with the increase in pectin, consistent with the results of Abid et al. [1]. The fruit has a more crumpled and soft skin. According to Culetu et al. [17], the presence of more skin in the jam helps to increase the hardness value. Therefore, the hardness will decrease with the increase in the mulberry content. Jams fractured more easily and became more brittle with increasing pectin concentrations [1]. All jam samples exhibited a maximum cohesiveness ranging from 0.84 to 0.89. The highest cohesion (0.89 ± 0.002) in the jams was observed at an *M. nigra* L. to *P. domestica* L. mass ratio of 5:0, while the lowest cohesiveness (0.84 ± 0.021) was observed at a ratio of 2:3.

### 3.2. Antioxidant Activity

As shown in Table 5, the total antioxidant capacity, hydroxyl radical scavenging capacity, DPPH radical scavenging capacity, and ABTS radical scavenging capacity of the jams tended to increase with increasing proportions of *M. nigra* L. Hao [33] demonstrated that the black mulberry exhibits a greater antioxidant capacity than that of other types of small berries and the strongest antioxidant capacity compared to other mulberries within the same genus. In the present study, the total polyphenol content of the jam also increased with increasing proportions of *M. nigra* L. Research shows that *P. domestica* L. has been shown to have a high content of phenolic compounds (111 mg/100 g) and carotenoids (1.3–2.3 mg/100 g). Phenolic compounds are prominent among the biologically active ingredients in mulberry, especially flavonoids, anthocyanins, and phenolic acids. Li et al. [34] reported a total phenol content of 192.67 mg GAE/g in *M. nigra* L. In the report by Erden [35], the total polyphenol content of *M. nigra* L. was measured at 192.67 mg GAE/g. It was observed that the polyphenol content in *M. nigra L.* was significantly higher than that in *P. domestica* L., leading to a significant increase in the polyphenol and antioxidant properties of the jam with the higher proportion of traditional Chinese mulberry.

### 3.3. Sensorial Profile

As shown in Table 6, among the sample groups, significantly higher sensory scores were observed at an *M. nigra* L. to *P. domestica* L. mass ratio of 4:1. Notably, the sensory texture score of the jam decreased as the proportion of *M. nigra* L. increased, which was associated with changes in hardness, adhesiveness, chewiness, and cohesiveness of the jam. The scores of the flavor and color parameters of jam increased first and then decreased. The highest average score was obtained when the jam sample with a mass ratio of *M. nigra* L. to *P. domestica* L. mass ratio of 4:1. However, there were no significant trends in the scores for odor and dispersibility parameters. The jam sample scored highest in sweetness and acidity attributes when the mass ratio of *M. nigra* L. to *P. domestica* L. was 4:1. The level of acidity is determined by the pH value: a lower pH level results in a more pronounced sour taste. The evaluators reported that the jam sample with a 5:0 mass ratio of *M. nigra* L. to *P. domestica* L. was overly sour, leading to a decrease in taste scores. As shown in Table 6, the total sensory scores tended to increase and then decrease with the increase in the proportion of *M. nigra* L. The highest total average sensory score was 77.90 at an *M. nigra* L. to *P. domestica* L. mass ratio of 4:1, followed by 77.68 at a ratio of 3:2, and 69.89 at a ratio of 2:3. The lowest sensory score of 59.08 was recorded at a mass ratio of 0:5. Similar results were obtained by Emelike and Akusu [36] for some tropical fruit jams and Nistor et al. [22] for buckthorn marmalades.

### 3.4. Metabolome

#### 3.4.1. Multivariate Statistical Analysis of Metabolome

**Figure 2:**
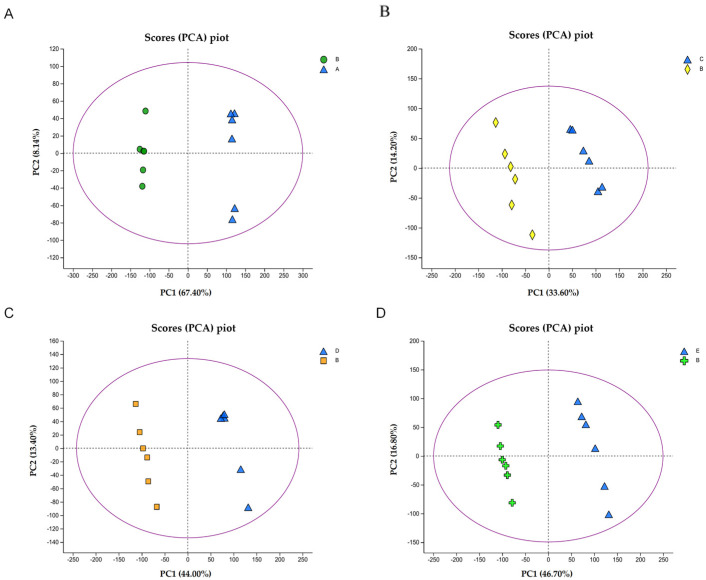
Plot of principal component analysis (PCA) scores of jams before and after thermal processing treatment and during storage: (**A**) plot of PCA scores of jams before and after thermal processing; (**B**) plot of PCA scores of jam storage day 0 vs. storage day 10; (**C**) plot of PCA scores of jam storage day 0 vs. storage day 20; (**D**) plot of PCA scores of jam storage day 0 vs. storage day 25.

The jam heat treatment and storage process involves five stages: A, B, C, D, and E. Group A represents the fruit samples before heat treatment, and group B represents fruit samples after heat treatment (jam samples on day 0 of storage). Groups C, D, and E correspond to jam samples on storage days 10, 20, and 25, respectively. A total of 1508 ion metabolite signatures were generated from these samples, including 821 positive ion patterns and 687 negative ion patterns. To reduce the dimensionality and increase the interpretability and validity of the data, multivariate statistical analysis techniques, including principal component analysis (PCA) and orthogonal partial least squares discriminant analysis (OPLS-DA) modeling, were applied to the processed metabolite lists for further analysis. The projected score values of the samples on the plane formed by the first principal component (PC1) and the second principal component (PC2) are the spatial coordinates, which can intuitively reflect the similarity or difference between the samples [5,37]. Dimensionality reduction analysis revealed relative coordinate points on the principal components p1 and p2. Moreover, the distance of each coordinate point represents the degree of aggregation and disaggregation between the samples, where shorter distances indicate higher similarity between the samples and longer distances indicate greater differences. As shown in Figure 2, a clear differentiation was observed among jams subjected to various treatments: jam samples before and after thermal processing treatment, and jam samples stored on days 0, 10, 20, and 25 were separated and specifically distinguished by PC1. Notably, the experiments had good stability and reproducibility. Moreover, the distribution results indicated that the metabolites differed significantly between samples and could be efficiently separated [37].

**Figure 3:**
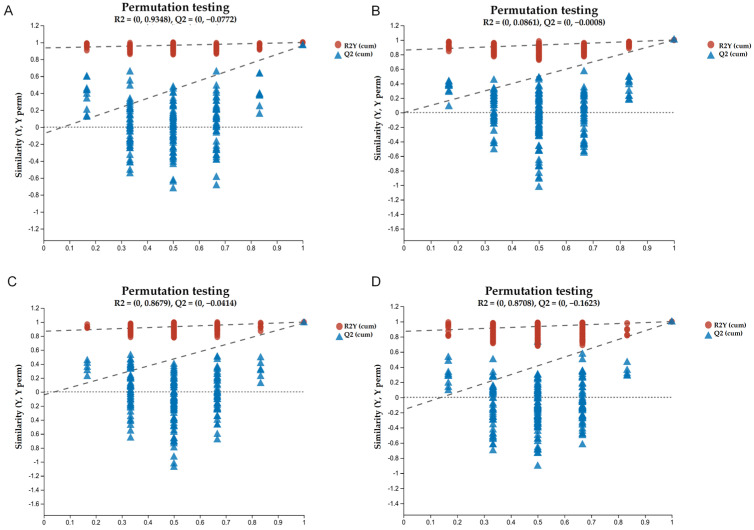
OPLS–DA scores of jams before and after thermal processing treatment and during storage. (**A**) OPLS–DA scores for jams before and after thermal processing; (**B**) OPLS–DA scores for jam on storage day 0 vs. storage day 10; (**C**) OPLS–DA scores for jam on storage day 0 vs. storage day 20; (**D**) OPLS–DA scores for jam storage on day 0 vs. storage day 25.

In general, the exact differences between samples cannot be explained by visual distinctions generated by PCA algorithms. Therefore, in this study, supervised classification was implemented via the OPLS-DA model to monitor the degree of metabolite transformation with thermal processing and storage. OPLS-DA can distinguish between two or more groups (classes) using multivariate data. The scoring plot output of the OPLS-DA multivariate approach is shown in Figure 3. Notably, the OPLS-DA scoring plot revealed a high degree of differentiation between sample groups, with a clear separation between the groups. As shown in Figure 3, the slopes of the Q2Y fitted regression lines for both sample groups of jam before and after heat treatment, as well as those during storage, all exceeded 1. Moreover, the detected values were all lower than the true values, and the intercept of the regression lines for Q2Y was less than 0.5, indicating that there was no overfitting in the established OPLS-DA model. The model parameters before and after thermal processing treatment were as follows: R2Y = 0.9348, Q2Y = 0.0772; storage days 0–10: R2Y = 0.861, Q2Y = 0.0008; day 20: R2Y = 0.8679, Q2Y = 0.0414; days 0–25: R2Y = 0.708, Q2Y = 0.1623. These results indicate that the OPLS-DA model is valid with good predictive ability and can be used to explore the metabolite differences during heat treatment and storage of composite jam [24].

#### 3.4.2. Screening for Differential Metabolites

The values VIP > 1 and *p* < 0.05 for principal component 1 (PCl) of the OPLS–DA model were used to identify potential biomarkers. In total, 219 differential metabolites were identified before and after heat treatment (185 up-regulated and 34 down-regulated); six differential metabolites were identified for day 0 vs. day 10 (six down-regulated), 49 differential metabolites were identified for day 0 vs. day 20 (forty-eight down-regulated, one up-regulated), and 83 differential metabolites were identified for day 0 vs. day 25 (two up-regulated and eighty-one down-regulated).

**Figure 4:**
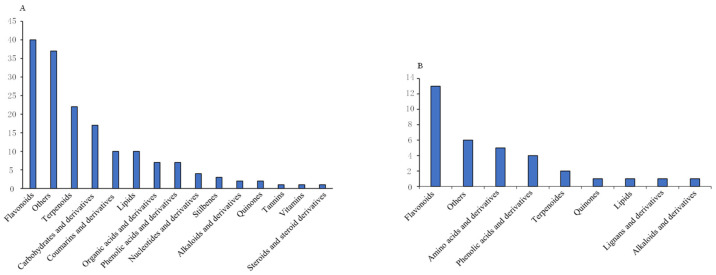
Differential metabolites of jam before and after thermal processing. (**A**) Up-regulated metabolite species; (**B**) down-regulated metabolite species.

To better understand the changes in the major differential metabolites before and after heat processing, we ranked the major differential metabolites detected by VIP markers (Figure 4) [37]. The results showed that the major differential metabolites before and after heat treatment of the jams were flavonoids, terpenoids, and carbohydrates, among which the down-regulated major differential metabolites were flavonoids, amino acids, and phenolic acids. The results suggest that these down-regulated metabolites were unstable during the heat treatment stage. More metabolites are destroyed or decomposed during the heating process than the amount that is increased during the concentration process. Feng et al. [38] demonstrated through experiments and multivariate statistical analysis that heat treatment had a significant impact on the metabolic components in black beans. The influence varied among different components, with polyphenolic compounds, particularly flavonoids and isoflavones, being highly susceptible.

**Figure 5:**
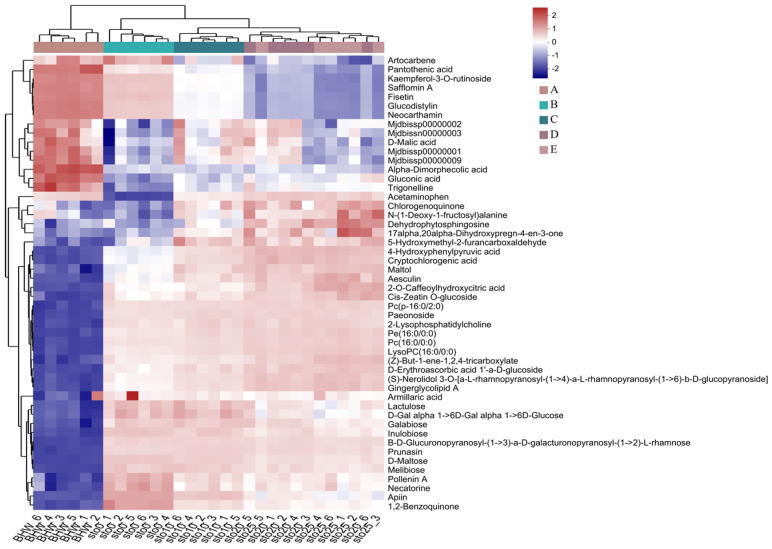
Cluster analysis of jam metabolites before and after thermal processing and during storage. BHW, sample group before thermal processing; sto0, sample group after thermal processing treatment (day 0 of storage); sto10, sample group on the 10th day of storage; sto20, sample group on the 20th day of storage; sto25, sample group on the 25th day of storage.

Notably, the metabolic profiles of primary metabolites showed relative differences before and after thermal processing and at different stages during storage. Differential metabolites were mainly expressed in terms of species and relative abundance, in parallel with which we established a cluster analysis (HCA) that revealed the diversity of metabolite profiles among different experimental samples. The resulting thermograms (Figure 5) clearly show different intensities of differential labeling between the jams before and after thermal processing and the jams with different storage times.

#### 3.4.3. Dynamic Changes in Polyphenols during Storage

One differential polyphenol metabolite (down-regulated) was identified from day 0 vs. day 10, 29 differential polyphenol metabolites (twenty-eight down-regulated, one up-regulated) were identified from day 0 vs. day 20, and 55 differential polyphenol metabolites (fifty-four down-regulated, one up-regulated) were identified from day 0 vs. day 25. This indicates that phenolic metabolites decreased during storage, and there were fewer changes during the first 10 days of storage, with only one metabolite significantly different; after 20 days of storage, the phenolics decreased more significantly. The specific phenolic compounds that declined are shown in Tables S1–S3. In addition to the down-regulated metabolites, one differentially up-regulated polyphenolic metabolite was identified during storage as arbutin; arbutin was differentially up-regulated at 20 days of storage, which is mainly related to glycolysis/glycolysis and carbohydrate metabolism [39].

### 3.5. Fragrance Change

#### 3.5.1. Comparative Analysis of Volatile Constituent Profiles in Single-Fruit and Mixed Jams

**Figure 6:**
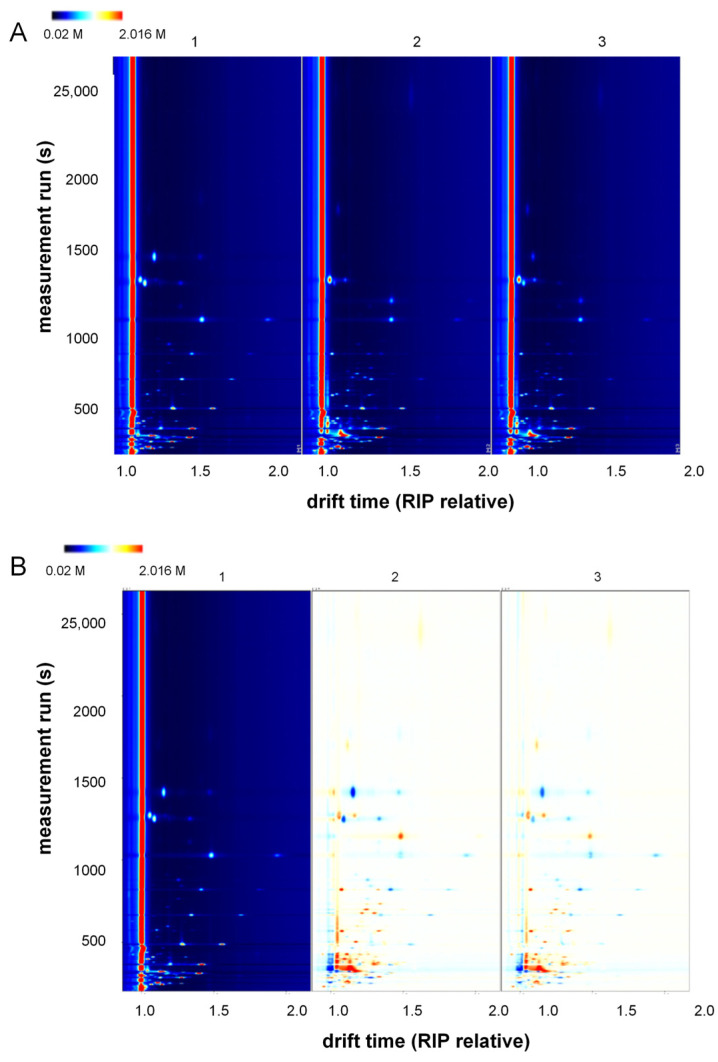
(**A**) Two-dimensional GC-IMS spectra of volatile components in the samples; (**B**) differential GC-IMS spectra of volatile components in the samples; (1) represents *Morus nigra* L. jam, (2) represents *Prunus domestica* L. jam, and (3) represents mixed jam.

Gas chromatography–ion mobility spectrometry (GC-IMS) was used to analyze various volatiles in samples of single-fruit jams and the mixed jams in this manuscript, yielding volatile organic compounds (VOCs) such as aldehydes, esters, alcohols, ketones, and others. The volatile organic compounds (VOCs) in the samples of *Morus nigra* L. jam, *Prunus domestica* L. jam, and their mixed jam were analyzed using GC-IMS. A top view of the analytical results is illustrated in Figure 6A, where several key features can be observed: (1) The background of the whole plot is blue, and the red vertical line at horizontal coordinate 1.0 is the RIP peak (reactive ion peak, normalized). (2) The vertical coordinate represents the retention time (s) of the GC, and the horizontal coordinate represents the relative migration time (normalized, a.u.). (3) Each point on either side of the RIP peak represents a VOC [40]. The color represents the peak intensity of the substance, from blue to red, with darker colors indicating greater peak intensity. There were some differences in the VOCs in different samples. To further visualize the differences in volatile components in the samples, the spectrum of a single *Morus nigra* L. jam sample 1 was selected as a reference, and the spectra of the other samples were subtracted from the reference to obtain a comparison of the differences in the different samples, as shown in Figure 6B. If the VOC contents in the target sample and the reference are the same, the background after deduction is white; the red color indicates that the concentration of the substance is higher in the target sample than in the reference, and the blue color indicates that the concentration of the substance is lower in the target sample than in the reference [26].

#### 3.5.2. Fingerprinting of Volatile Components in Single-Fruit and Mixed Jams

**Figure 7:**
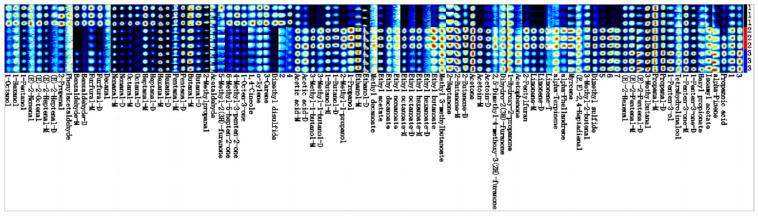
Fingerprints of volatile components in the samples; The brighter colors in the figure represent higher concentrations, with red indicating a higher concentration than blue; (1) represents *Morus nigra* L. jam, (2) represents *Prunus domestica* L. jam, and (3) represents mixed jam.

To accurately evaluate closely related substances on the topographic map, all information provided by the fingerprinting technique was utilized for qualitative characterization. In the fingerprint map (Figure 7), each row displays all signal peaks of the sample, while each column represents the same volatile compounds in different samples [41]. Additionally, their colors correspond to the content of volatile compounds, with brighter shades indicating higher concentrations. As depicted in Figure 7, there were significant variations in VOC contents among each group of samples. A total of 68 volatile components with 90 peaks were identified across the three samples, and unidentified VOCs are denoted by numbers 1–7. The identified compounds were categorized into six groups comprising twenty-one aldehydes, twelve esters, twelve alcohols, eleven ketones, seven alkenes, and seven other compounds [41].

As shown in Figure 7, there were significant differences between *Morus nigra* L. jam and mixed jam, and smaller differences between *Prunus domestica* L. jam and mixed jam. The contents of volatile organic compounds were similar, including volatiles such as Hexyl propionate; Tetrahydrolinalool; 1-Penten-3-ol, 2-Methylbutanal; Pentanal with a fruity aroma; 1-Penten-3-one with a spicy, etheric, peppery, garlicky, mustardy, and oniony aroma; Butanal with an ethereal freshness when very diluted; and Hydroxy-2-propanone with a caramelized aroma. Compared with the other samples, 1-Octanol, 1-Hexanol, Phenylacetaldehyde, Benzaldehyde, Furfural, Decanal, Nonanal, Octanal, Heptanal, Heptanal, and Phenylacetaldehyde were the most common odors in the *Morus nigra* L. jam. Compared to other samples, *Morus nigra* L. 1-Octanol, 1-Hexanol, Phenylacetaldehyde, Benzaldehyde, Furfural, Decanal, Nona-nal, Octanal, Heptana, Pentanal, Butanal, Dihydro-2(3H)-furanone, 6-Methyl-5-hepten-2-one, 4-Methyl-3-penten-2-one, 1-Octen-3-one, and 3-Carene had a higher content of volatile aroma components. Compared to the other samples, *Prunus domestica* L. jam contained more volatile aroma components than the other jams, including 2-Methyl-1-butanol, 1-Butanol, Ethanol, Methyl decanoate, Ethyl acetate, Ethyl decanoate, Ethyl Ethyl decanoate, Ethyl acetate, Ethyl decanoate, Ethyl nonanoate, Decanal, Ethyl hexanoate, Ethyl butanoate, 2-Heptanone, 2-Butanone, Acetone, Acetoin, 2,5-Dimethyl-4-methoxy-3(2H)-furanone, Acetophenone, Pentoxy-3(2H)-furanone, Acetophenone, Pentosol, Acetophenone, Acetophenone, Acetophenone, Pentylfuran, Limonene, alpha-Terpinene, alpha-Phellandrene, Myrcene, and (E, E)-2,4-Heptadienal. Compared to the other samples, the mixed jam contained more volatile aroma components than the single-fruit jam, including (E)-2-Hexenal, (E)-2-Pentenal, 3-methylbutanal, Propanal, 1-Penten-3-ol, Tetrahydrolinalool, 1-Penten-3-one, Isoamyl acetate, and alpha-Pinene, which were found in the mixed jam. The aromas of the volatile components mentioned above are shown in Table S4.

## 4. Conclusions

In the initial study, we examined the composite jam formula of Hesse fruit and plums and determined the optimal fruit ratio to be 4:1 (*w*/*w*). UHPLC-Q results revealed 219 different metabolites identified before and after heat treatment, with the different metabolites mainly consisting of flavonoids, terpenes, and carbohydrates. Key flavonoids, amino acids, and phenolic acids were down-regulated. During storage, a total of eighty-three different metabolites were identified, with two up-regulated and eighty-one down-regulated, mostly polyphenols. The results of GC-IMS showed that the mixed jam has a volatile aroma component similar to *Prunus domestica* L. jams. Mixed jams contain high levels of various aromatic compounds, including hexenal, pentenal, 3-methylbutyraldehyde, 1-pentenol, tetrahydrolinalsol, 1-hexylpropionic acid, isoamyl acetate, alpha-pentene, and propionic acid, which contribute to the pleasing aroma of jams, similar to that of vanilla fruits. The techniques of GC-IMS, metabolomics, and the apparent indicators utilized in this study will provide a comprehensive and in-depth evaluation system. This system can be used as a framework to explore the development of other composite foods or to further optimize the formulation and create new food products that align with consumer preferences.

## Figures and Tables

**Table 1:** Sensory evaluation criteria for mixed jam.

Item	Trait Performance	Score
Spreadability (15 points)	Easy to apply, even, continuous, smooth coating, no faults	10.1–15
	Easier to apply, more even coating, some consistency	5.1–10
	Not suitable for application, uneven coating, not easy to push away	0–5
Odor (15 points)	Rich in odor, with fruity notes of *Morus nigra* L. or *Prunus domestica* L.	10.1–15
	*Morus nigra* L. or *Prunus domestica* L. are less fruity	6.1–10
	Strong irritating odor, no *Morus nigra* L. or *Prunus domestica* L. fruit aroma	0–6
Texture (20 points)	Good gelation, no layering, no liquid precipitation on the surface	15.1–20
	Basic gel formation, flow feeling, slight water precipitation	8.1–15
	No gel formation, strong flow sensation, layering phenomenon	0–8
Color (20 points)	Uniform color and high color brightness	15.1–20
	More uniform color, lower color brightness	8.1–15
	Uneven color, dark color brightness	0–8
Taste (30 points)	Moderately tart or sweet, fruity, and smooth	20.1–30
	Tart or sweet, less fruity, and smoother in texture	10.1–20
	Excessive acidity or sweetness, off-flavor, sticky and uneven texture	0–10

**Table 2:** pH and total acid of mixed jam.

*Morus nigra* L. to *Prunus domestica* L. in Jam (*w*/*w*)	0:5	1:4	2:3	3:2	4:1	5:0
pH	4.13 ± 0.005 ^a^	3.99 ± 0.015 ^b^	3.84 ± 0.050 ^c^	3.61 ± 0.010 ^d^	3.41 ± 0.006 ^e^	3.25 ± 0.006 ^f^
Total acid (%)	3.30 ± 0.004 ^f^	6.74 ± 0.005 ^e^	10.45 ± 0.007 ^d^	13.62 ± 0.004 ^c^	15.37 ± 0.002 ^b^	18.09 ± 0.003 ^a^

Significant differences (*p* < 0.05) in the same column are indicated by different lower case letters.

**Table 3:** Effect of fruit pulp mixing ratio on color characteristics of jam.

*Morus nigra* L. to *Prunus domestica* L. in Jam (*w*/*w*)	L*	a*	b*	∆E
0:5	31.85 ± 0.03 ^a^	2.51 ± 0.10 ^c^	2.56 ± 0.33 ^a^	68.15 ± 0.04 ^b^
1:4	29.37 ± 0.20 ^b^	3.37 ± 0.04 ^ab^	−0.16 ± 0.31 ^b^	70.63 ± 0.20 ^a^
2:3	28.73 ± 0.13 ^bc^	3.45 ± 0.11 ^a^	−0.54 ± 0.26 ^b^	71.27 ± 0.13 ^a^
3:2	28.70 ± 0.06 ^bc^	3.09 ± 0.41 ^d^	−1.00 ± 0.16 ^c^	71.30 ± 0.05 ^a^
4:1	28.73 ± 0.11 ^bc^	3.03 ± 0.19 ^b^	−1.01 ± 0.22 ^c^	71.27 ± 0.13 ^a^
5:0	28.14 ± 1.58 ^c^	2.28 ± 0.02 ^cd^	−1.01 ± 0.06 ^c^	71.86 ± 1.56 ^a^

Significant differences (*p* < 0.05) in the same column are indicated by different lowercase letters.

**Table 4:** Effect of fruit pulp ratio on texture characteristics of jam.

*Morus nigra* L. to *Prunus domestica* L. in Jam (*w*/*w*)	Hardness	Adhesiveness	Chewiness	Cohesiveness
0:5	47.76 ± 0.75 ^a^	−726.58 ± 21.17 ^a^	36.70 ± 1.39 ^a^	0.84 ± 0.021 ^b^
1:4	43.34 ± 2.06 ^b^	−603.90 ± 2.76 ^b^	33.23 ± 2.34 ^b^	0.83 ± 0.006 ^b^
2:3	38.20 ± 0.94 ^c^	−473.91 ± 12.99 ^c^	27.17 ± 0.74 ^c^	0.80 ± 0.002 ^c^
3:2	36.66 ± 0.92 ^cd^	−375.14 ± 6.37 ^d^	26.83 ± 1.25 ^c^	0.81 ± 0.013 ^bc^
4:1	34.56 ± 1.66 ^e^	−351.49 ± 0.78 ^e^	25.85 ± 1.88 ^c^	0.83 ± 0.013 ^bc^
5:0	19.10 ± 0.73 ^f^	−29.59 ± 2.83 ^f^	14.38 ± 0.78 ^d^	0.89 ± 0.002 ^a^

Significant differences (*p* < 0.05) in the same column are indicated by different lowercase letters.

**Table 5:** Effects of fruit pulp ratio on antioxidant and total polyphenols.

*Morus nigra* L. to *Prunus domestica* L. in Jam (*w*/*w*)	Total Antioxidant Capacity (U/mL)	OH (%)	DPPH (mM TE /g FW)	ABTS (mM TE/g FW)	Total Polyphenol (mgGAE/100 g FW)
0:5	205.73 ± 12.58 ^e^	0.08 ± 0.002 ^d^	0.84 ± 0.021 ^b^	2.77 ± 0.27 ^d^	381.00 ± 26.60 ^d^
1:4	229.89 ± 8.15 ^d^	0.08 ± 0.006 ^d^	0.83 ± 0.006 ^b^	3.35 ± 0.29 ^d^	403.35 ± 2.34 ^cd^
2:3	250.86 ± 1.48 ^c^	0.09 ± 0.004 ^cd^	0.80 ± 0.002 ^c^	4.68 ± 0.31 ^c^	422.25 ± 3.75 ^c^
3:2	265.81 ± 13.70 ^c^	0.10 ± 0.008 ^bc^	0.81 ± 0.013 ^bc^	5.46 ± 0.87 ^c^	428.95 ± 7.46 ^c^
4:1	319.95 ± 5.92 ^b^	0.11 ± 0.005 ^b^	0.83 ± 0.013 ^bc^	6.67 ± 0.41 ^b^	550.40 ± 13.76 ^b^
5:0	375.55 ± 14.43 ^a^	0.12 ± 0.009 ^a^	0.89 ± 0.002 ^a^	8.08 ± 0.38 ^a^	649.31 ± 7.37 ^a^

Significant differences (*p* < 0.05) in the same column are indicated by different lowercase letters.

**Table 6:** Effect of fruit pulp ratio on sensory score of jam.

*Morus nigra* L. to *Prunus domestica* L. in Jam (*w*/*w*)	Spreadability	Odor	Texture	Color	Taste	Total Sensory Score
0:5	8.88 ± 2.57 ^b^	11.53 ± 2.91 ^a^	17.37 ± 0.82 ^a^	8.45 ± 2.11 ^c^	12.45 ± 4.51 ^d^	59.08 ± 10.74 ^c^
1:4	9.30 ± 2.58 ^b^	11.48 ± 2.76 ^a^	17.45 ± 0.69 ^a^	11.63 ± 2.64 ^b^	15.62 ± 4.59 ^cd^	65.48 ± 9.10 ^bc^
2:3	10.08 ± 2.52 ^ab^	11.40 ± 2.12 ^a^	18.22 ± 0.63 ^a^	14.72 ± 2.31 ^a^	15.22 ± 2.14 ^cd^	69.63 ± 5.66 ^ab^
3:2	12.30 ± 2.98 ^a^	10.77 ± 3.19 ^a^	18.02 ± 0.67 ^a^	15.62 ± 1.42 ^a^	20.98 ± 4.62 ^ab^	77.68 ± 9.95 ^a^
4:1	11.22 ± 1.99 ^ab^	12.33 ± 2.14 ^a^	14.77 ± 1.03 ^b^	16.62 ± 1.17 ^a^	22.97 ± 3.71 ^a^	77.90 ± 6.69 ^a^
5:0	11.03 ± 1.49 ^ab^	11.70 ± 3.16 ^a^	10.73 ± 2.38 ^c^	15.57 ± 2.01 ^a^	19.02 ± 4.82 ^bc^	68.05 ± 10.12 ^b^

Significant differences (*p* < 0.05) in the same column are indicated by different lowercase letters.

## Data Availability

The original contributions presented in the study are included in the article and Supplementary Material, further inquiries can be directed to the corresponding author.

## Supplementary Materials

The following supporting information can be downloaded at: https://www.mdpi.com/article/10.3390/foods13071104/s1, Table S1: Differential polyphenol metabolites down-regulated in the comparison of day 0 and day 10 jam samples; Table S2: Differential polyphenol metabolites down-regulated in the comparison between day 0 and day 20 jam samples; Table S3: Differential polyphenol metabolites down-regulated in the comparison of day 0 and day 25 jam samples; Table S4: Odor descriptions of some volatile aroma components.
