# Supplementary material for: A Novel Strategy for Mixed Jam Evaluation: Apparent Indicator, Sensory, Metabolomic, and GC-IMS Analysis
Source: Foods. 2024 Apr 3;13(7):1104. doi: 10.3390/foods13071104 (PMC11011859; doi:10.3390/foods13071104)
Supplement: Supplementary file 1 [file foods-13-01104-s001.zip › Table S1.pdf]

Table S1. Differential polyphenol metabolites down-regulated in the comparison of day 0 and day 10 jam samples.

NO.	Metabolite	Formula	Class I	CAS ID
1	Epigallocatechin gallate	$C_{22}H_{18}O_{11}$	Flavonoids	989-51-5
