# Supplementary material for: A Novel Strategy for Mixed Jam Evaluation: Apparent Indicator, Sensory, Metabolomic, and GC-IMS Analysis
Source: Foods. 2024 Apr 3;13(7):1104. doi: 10.3390/foods13071104 (PMC11011859; doi:10.3390/foods13071104)
Supplement: Supplementary file 1 [file foods-13-01104-s001.zip › Table S2.pdf]

Table S2. Differential polyphenol metabolites down-regulated in the comparison between day 0 and day 20 jam samples.

NO.	Metabolite	Formula	Class I	CAS ID
1	Cubebinone	$C_{23}H_{26}O_8$	Lignans and derivatives	101751-71-7
2	Epigallocatechin gallate	$C_{22}H_{18}O_{11}$	Flavonoids	989-51-5
3	Quercetin 3-glucoside 7-xyloside	$C_{26}H_{28}O_{16}$	Flavonoids	79592-61-3
4	Feruloyl C1-glucuronide	$C_{16}H_{18}O_{10}$	Phenolic acids and derivatives	-
5	Limocitrin 3-rhamnoside	$C_{23}H_{24}O_{12}$	Flavonoids	90456-56-7
6	Isovitexin 2''-O-glucoside	$C_{27}H_{30}O_{15}$	Flavonoids	60767-80-8
7	Astilbin	$C_{21}H_{22}O_{11}$	Flavonoids	29838-67-3
8	Kuwanon Z	$C_{34}H_{26}O_{10}$	Flavonoids	104931-21-7
9	Luteoforol	$C_{15}H_{14}O_6$	Flavonoids	24897-98-1
10	2'-O-Methylisoliquiritigenin	$C_{16}H_{14}O_4$	Flavonoids	112408-67-0
11	Isovitexin 2''-(6'''-p-coumaroylglucoside)	$C_{36}H_{36}O_{17}$	Flavonoids	-
12	Dihydroisorhamnetin	$C_{16}H_{14}O_7$	Flavonoids	55812-91-4
13	2,4,6-Trihydroxybenzoic acid	$C_7H_6O_5$	Phenolic acids and derivatives	83-30-7
14	Pipecolic acid betaine	$C_{21}H_{20}O_{10}$	Flavonoids	472-22-0
15	Isorhamnetin 3-(6''-malonylglucoside)	$C_{25}H_{24}O_{15}$	Flavonoids	86555-37-5
16	2'-Hydroxygenistein	$C_{15}H_{10}O_6$	Flavonoids	1156-78-1
17	3-Galloylgallocatechin	$C_{22}H_{18}O_{11}$	Flavonoids	5127-64-0
18	5,7,3'-Trihydroxy-4'-methoxyflavanone	$C_{16}H_{14}O_6$	Flavonoids	520-33-2
19	Glyceollin III	$C_{20}H_{18}O_5$	Flavonoids	61080-23-7
20	Naringin	$C_{27}H_{32}O_{14}$	Flavonoids	10236-47-2
21	Tricetin	$C_{15}H_{10}O_7$	Flavonoids	520-31-0
22	3'-Hydroxygenistein	$C_{15}H_{10}O_6$	Flavonoids	-
23	Capillarisin	$C_{16}H_{12}O_7$	Coumarins and derivatives	56365-38-9
24	Quercetin 3-O-rhamnoside	$C_{21}H_{20}O_{11}$	Flavonoids	522-12-3
25	(-)-Epigallocatechin	$C_{15}H_{14}O_7$	Flavonoids	970-74-1
26	Medicocarpin	$C_{22}H_{24}O_9$	Flavonoids	52766-70-8
27	Hovenitin I	$C_{16}H_{14}O_8$	Flavonoids	71106-82-6
28	Alpha-(1,2-Dihydroxyethyl)-1,2,3,4-tetrahydro-7-hydroxy-9-methoxy-3,4-dioxocyclopenta[c][1]benzopyran-6-acetaldehyde	$C_{17}H_{16}O_8$	Coumarins and derivatives	-
