# Supplementary material for: A Novel Strategy for Mixed Jam Evaluation: Apparent Indicator, Sensory, Metabolomic, and GC-IMS Analysis
Source: Foods. 2024 Apr 3;13(7):1104. doi: 10.3390/foods13071104 (PMC11011859; doi:10.3390/foods13071104)
Supplement: Supplementary file 1 [file foods-13-01104-s001.zip › Table S3.pdf]

**Table S3.** Differential polyphenol metabolites down-regulated in the comparison of day 0 and day 25 jam samples.

| NO. | Metabolite | Formula | Class I | CAS ID |
|---|---|---|---|---|
| 1 | Cubebinone | $C_{23}H_{26}O_8$ | Lignans and derivatives | 101751-71-7 |
| 2 | 2'-Hydroxygenistein 7-(6"-malonylglucoside) | $C_{24}H_{22}O_{14}$ | Flavonoids | 137351-13-4 |
| 3 | Epigallocatechin gallate | $C_{22}H_{18}O_{11}$ | Flavonoids | 989-51-5 |
| 4 | Catechin 3'-glucuronide | $C_{21}H_{22}O_{12}$ | Flavonoids | 853728-25-3 |
| 5 | Quercetin 3-glucoside 7-xyloside | $C_{26}H_{28}O_{16}$ | Flavonoids | 79592-61-3 |
| 6 | Feruloyl C1-glucuronide | $C_{16}H_{18}O_{10}$ | Phenolic acids and derivatives | - |
| 7 | Kaempferol-3-O-Arabinofuranoside | $C_{20}H_{18}O_{10}$ | Flavonoids | - |
| 8 | Limocitrin 3-rhamnoside | $C_{23}H_{24}O_{12}$ | Flavonoids | 90456-56-7 |
| 9 | (+)-taxifolin | $C_{15}H_{12}O_7$ | Flavonoids | 480-18-2 |
| 10 | Neocarthamin | $C_{21}H_{22}O_{11}$ | Flavonoids | - |
| 11 | Cichoriin | $C_{15}H_{16}O_9$ | Coumarins and derivatives | 531-58-8 |
| 12 | Serotonin | $C_{10}H_{12}N_2O$ | Indoles and derivatives | 50-67-9 |
| 13 | 2,4',5,7-Tetrahydroxyflavanone | $C_{15}H_{12}O_6$ | Flavonoids | 58124-18-8 |
| 14 | Daidzein 7-O-glucuronide | $C_{21}H_{18}O_{10}$ | Flavonoids | - |
| 15 | Conidendrin | $C_{20}H_{20}O_6$ | Lignans and derivatives | 518-55-8 |
| 16 | Isovitexin 2"-O-glucoside | $C_{27}H_{30}O_{15}$ | Flavonoids | 60767-80-8 |
| 17 | Diospyrin | $C_{21}H_{24}O_{13}$ | Flavonoids | 27838-81-9;28164-57-0 |
| 18 | Isoeriocitrin | $C_{27}H_{32}O_{15}$ | Flavonoids | - |
| 19 | Astilbin | $C_{21}H_{22}O_{11}$ | Flavonoids | 29838-67-3 |
| 20 | Kuwanon Z | $C_{34}H_{26}O_{10}$ | Flavonoids | 104931-21-7 |
| 21 | Luteoforol | $C_{15}H_{14}O_6$ | Flavonoids | 24897-98-1 |
| 22 | 2'-O-Methylisoliquiritigenin | $C_{16}H_{14}O_4$ | Flavonoids | 112408-67-0 |
| 23 | 4-Methylumbelliferone glucuronide | $C_{16}H_{16}O_9$ | Coumarins and derivatives | 6160-80-1 |
| 24 | Isovitexin 2"-(6"'-p-coumaroylglucoside) | $C_{36}H_{36}O_{17}$ | Flavonoids | - |
| 25 | Neoeriocitrin | $C_{27}H_{32}O_{15}$ | Flavonoids | 13241-32-2 |
| 26 | Daidzein 4'-O-glucuronide | $C_{21}H_{18}O_{10}$ | Flavonoids | - |
| 27 | Dihydroisorhamnetin | $C_{16}H_{14}O_7$ | Flavonoids | 55812-91-4 |
| 28 | L-Epicatechol | $C_{15}H_{14}O_6$ | Flavonoids | 490-46-0 |
| 29 | Kaempferol 3-O-arabinoside | $C_{20}H_{18}O_{10}$ | Flavonoids | - |
| 30 | 2,4,6-Trihydroxybenzoic acid | $C_7H_6O_5$ | Phenolic acids and derivatives | 83-30-7 |
| 31 | Pipecolic acid betaine | $C_{21}H_{20}O_{10}$ | Flavonoids | 472-22-0 |

| | | | | |
|---|---|---|---|---|
| 32 | 3,7-Dimethylquercetin | $C_{17}H_{14}O_7$ | Flavonoids | 2068/2/2 |
| 33 | 2'-Hydroxygenistein | $C_{15}H_{10}O_6$ | Flavonoids | 1156-78-1 |
| 34 | 3-Galloylgallocatechin | $C_{22}H_{18}O_{11}$ | Flavonoids | 5127-64-0 |
| 35 | 5,7,3'-Trihydroxy-4'-methoxyflavanone | $C_{16}H_{14}O_6$ | Flavonoids | 520-33-2 |
| 36 | Glyceollin III | $C_{20}H_{18}O_5$ | Flavonoids | 61080-23-7 |
| 37 | Scolymoside | $C_{27}H_{30}O_{15}$ | Flavonoids | 25694-72-8 |
| 38 | Catechin 7-glucuronide | $C_{21}H_{22}O_{12}$ | Flavonoids | 1146696-34-5 |
| 39 | Maesopsin 6-glucoside | $C_{21}H_{22}O_{11}$ | Flavonoids | - |
| 40 | Naringin | $C_{27}H_{32}O_{14}$ | Flavonoids | 10236-47-2 |
| 41 | Melitric acid B | $C_{27}H_{20}O_{11}$ | Phenolic acids and derivatives | 153765-46-9 |
| 42 | Bergapten | $C_{12}H_8O_4$ | Coumarins and derivatives | 484-20-8 |
| 43 | Tricetin | $C_{15}H_{10}O_7$ | Flavonoids | 520-31-0 |
| 44 | 3'-Hydroxygenistein | $C_{15}H_{10}O_6$ | Flavonoids | - |
| 45 | Capillarisin | $C_{16}H_{12}O_7$ | Coumarins and derivatives | 56365-38-9 |
| 46 | Quercetin 3-O-rhamnoside | $C_{21}H_{20}O_{11}$ | Flavonoids | 522-12-3 |
| 47 | (-)-Epigallocatechin | $C_{15}H_{14}O_7$ | Flavonoids | 970-74-1 |
| 48 | Dattelic acid | $C_{16}H_{16}O_8$ | Phenolic acids and derivatives | 73263-62-4 |
| 49 | Medicocarpin | $C_{22}H_{24}O_9$ | Flavonoids | 52766-70-8 |
| 50 | Kaempferol 3-xylosylglucoside | $C_{26}H_{28}O_{15}$ | Flavonoids | 29322-07-4 |
| 51 | Hovenitin I | $C_{16}H_{14}O_8$ | Flavonoids | 71106-82-6 |
| 52 | Alpha-(1,2-Dihydroxyethyl)-1,2,3,4-tetrahydro-7-hydroxy-9-methoxy-3,4-dioxocyclopenta[c][1]benzopyran-6-acetaldehyde | $C_{17}H_{16}O_8$ | Coumarins and derivatives | - |
| 53 | Aflatoxin B1 diol | $C_{17}H_{14}O_8$ | Coumarins and derivatives | 50668-79-6 |
| 54 | Epicatechin | $C_{15}H_{14}O_6$ | Flavonoids | 490-46-0;154-23-4 |
