# Supplementary material for: A Novel Strategy for Mixed Jam Evaluation: Apparent Indicator, Sensory, Metabolomic, and GC-IMS Analysis
Source: Foods. 2024 Apr 3;13(7):1104. doi: 10.3390/foods13071104 (PMC11011859; doi:10.3390/foods13071104)
Supplement: Supplementary file 1 [file foods-13-01104-s001.zip › Table S4.pdf]

Table S4. Odour descriptions of some volatile aroma components.

NO.	Metabolite	Flavor Profile
1	Hexyl propionate	Pear, green, fruity, musty, earthy and spicy.
2	1-Penten-3-one	Strong pungent odour of spicy, etheric, peppery, garlic, mustard and onion.
3	Tetrahydrolinaloo	Fresh Sweet Citrus Lime Floral Lily
4	1-Penten-3-ol	ethereal horseradish green radish chrysanthemum vegetable tropical fruity
5	2-Methylbutanal	Smells like apples
6	Butanal	Extremely diluted, it has an ethereal aroma.
7	Pentanal	Fermented Bread Flavour Fruity Flavour
8	1-Hydroxy-2-propanone	Caramel aroma
9	1-Octanol	It has a dry, sweet and sharp aroma of tallow and wax, with citrus, orange peel and rose-like notes, and a creamy base like anthemis root, which does not last long. It is also fruity, sweet and slightly grassy.
10	1-Hexanol	Aromas of young green leaves, with hints of wine, fruit and fat.
11	Phenylacetaldehyde	Aroma similar to hyacinth, with sweet fruity notes when diluted.
12	Benzaldehyde	Like almonds.
13	Furfural	Smells like almonds.
14	Decanal	Strong citrus peel odour
15	Nonanal	It has a strong greasy note, and when diluted it shows rose and citrus-like aromas.
16	Octanal	Strong fruity flavour
17	Heptanal	Fresh, green, sweet herbaceous odour.
18	Pentanal	Fermented Bread Flavour Fruity Flavour
19	Butanal	ethereal aldehydic chocolate peach fatty
20	Dihydro-2(3H)-furanone	Aromatic mint flavour.
21	6-Methyl-5-hepten-2-one	Citrus, Fruity
22	4-Methyl-3-penten-2-one	Smells of mint or honey
23	1-Octen-3-one	Strong loamy, milled mushroom aroma and metallic notes.
24	3-Carene	Smells like turpentine.
25	1-Methyl-1-butanol	Misty miscellaneous alcohol Alcohol Fatty greasy alcoholic flavour Whisky Leather Cocoa
26	1-Butanol	Has a peculiar alcohol odour
27	Ethanol	Smells like wine.
28	Methyl decanoate	Oily Wine Fruity Floral

29	Ethyl acetate	oily wine fruity floral
30	Ethyl decanoate	Fruity and winey aromas, pear and brandy-like notes.
31	Ethyl nonanoate	Intense aroma of grapes, with rose, fruity and winey notes.
32	Decanal	Strong aldehydic aroma, with sweet orange and tangerine aromas when diluted, with greasy notes.
33	Ethyl hexanoate	Has a pleasant odour
34	Ethyl butanoate	Pineapple flavour
35	2-Heptanone	Has a fruity, pear-like flavour
36	Acetone	Aromatic odour, highly volatile
37	Acetoin	Has a pleasant creamy flavour
38	2,5-Dimethyl-4-methoxy-3(2H)-furanone	Bread Crust, Butter, Caramel, Floral, Fruit
39	Acetophenone	Almonds, Flower, Meat, Must
40	2-Pentylfuran	Butter, Floral, Fruit, Green Bean
41	Limonene	Citrus, Mint
42	alpha-Terpinene	Refreshing lemon citrus aroma
43	alpha-Phellandrene	Aroma of black pepper and amygdala.
44	Myrcene	Has a special natural aroma, similar to that of bay leaves.
45	(E,E)-2,4-Heptadienal	Possesses a special aromatic odour
46	2-Methyl-2-butenal	Almond, mild butter aroma
47	(E)-2-Hexenal	Intense fruity, green aroma, vegetable aroma
48	(E)-2-Pentenal	Pungent green, fruity
49	3-methylbutanal	Fruity, fatty, animal and almond flavours.
50	Propanal	Strong fruity flavour
51	1-Penten-3-ol	Strong grass-green odour
52	Tetrahydrolinalool	fruity
53	Isoamyl acetate	Smells like bananas.
54	alpha-Pinene	Turpentine odour
